# Differential gene transcription across the life cycle in *Daphnia magna* using a new all genome custom-made microarray

**DOI:** 10.1186/s12864-018-4725-7

**Published:** 2018-05-18

**Authors:** Bruno Campos, Danielle Fletcher, Benjamín Piña, Romà Tauler, Carlos Barata

**Affiliations:** 1 IDAEA-CSIC: Institute of Environmental Diagnosis and Water Research, CSIC, Barcelona, Spain; 2 Agilent Technologies, Brighton, UK

**Keywords:** Transcription, Microarray, Development, Daphnia, Ecotoxicogenomics, Moult, Functional annotation

## Abstract

**Background:**

Unravelling the link between genes and environment across the life cycle is a challenging goal that requires model organisms with well-characterized life-cycles, ecological interactions in nature, tractability in the laboratory, and available genomic tools. Very few well-studied invertebrate model species meet these requirements, being the waterflea *Daphnia magna* one of them. Here we report a full genome transcription profiling of *D. magna* during its life-cycle. The study was performed using a new microarray platform designed from the complete set of gene models representing the whole transcribed genome of *D. magna.*

**Results:**

Up to 93% of the existing 41,317 *D. magna* gene models showed differential transcription patterns across the developmental stages of *D. magna*, 59% of which were functionally annotated. Embryos showed the highest number of unique transcribed genes, mainly related to DNA, RNA, and ribosome biogenesis, likely related to cellular proliferation and morphogenesis of the several body organs. Adult females showed an enrichment of transcripts for genes involved in reproductive processes. These female-specific transcripts were essentially absent in males, whose transcriptome was enriched in specific genes of male sexual differentiation genes, like *doublesex*.

**Conclusion:**

Our results define major characteristics of transcriptional programs involved in the life-cycle, differentiate males and females, and show that large scale gene-transcription data collected in whole animals can be used to identify genes involved in specific biological and biochemical processes.

**Electronic supplementary material:**

The online version of this article (10.1186/s12864-018-4725-7) contains supplementary material, which is available to authorized users.

## Background

Unravelling the link between genes and environment across a life-cycle is a challenging goal. The full exploration of this link requires model organisms with well-characterized ecological interactions in nature, tractability in the laboratory and available genomic tools [1]. In *Drosophila*, the vast majority of genes are transcribed during the first embryonic stages due to the contribution of both maternally-inherited and zygote-produced mRNAs, while adult females and males display specific transcribed genes, consistent with the known transcriptional profiles of ovaries and testis [2, 3]. Embryogenesis in the worm *Caenorhabditis elegans* elicits major transcriptional changes, particularly during the early and late stages, with a significant lower number of gene expression changes in mid-embryogenesis [4]. Males of *C. elegans* show a large number of highly expressed, male-specific genes, most of which being undetectable in any hermaphrodite stage [4]. Similar transcription gene patterns during embryogenesis and adult stages have been reported for many other species of sponges and insects [5, 6]. In the above mentioned species, the observed high transcription of genes during embryogenesis are associated with morphogenesis, differentiation, and development, and larval or adult stages show different clusters of gene transcription often related with particular tissues and organs.

The waterflea *Daphnia magna* Strauss is a very useful species for both environmental and basic biology studies [7, 8]. *D. magna* can be found in freshwater ecosystems all around the world, playing a relevant ecological role in the food web, both as a primary consumer and as a food source for predators, both vertebrates and invertebrates [9]. *D. magna* can be maintained in the lab as genetically uniform clonal lines through asexual parthenogenic reproduction, hereby reducing genetic variability and enabling the parallel analysis of functional and fitness changes in the same genotype in multiple environmental conditions [10]. *D. magna* and its close relative *D. pulex* are widely used as indicators of environmental quality [11], as models in evolutionary biology, and in the study of adaptive responses to environmental changes [12–16].

Daphnids genomes have recently received much attention from the scientific community, with the publication of two full genomes (*D. pulex* and *D. magna)* and two more species having their genomes partially sequenced [1, 17–20]. The specific function of many genes, however, even in well studied organisms, is incomplete. It is not unusual to find a third of the genes in any genome to lack functional annotation [21, 22]. This lack of functional annotation is higher in newly sequenced organisms that often contain lineage-specific genes lacking homology with other assembled genomes [22, 23]. This is the case for *D. magna* and its close relative *D. pulex*, [1, 20, 23, 24]. In *D. pulex*, one third of its genome comprises lineage-specific genes that lack orthologs in other eukaryote genomes and thus lack any functional annotation [20], and the situation is probably similar for the *D. magna* genome [17]. Transcriptomic studies in *D. magna* and related species have been up to now mostly conducted to evaluate the response of genes to environmental factors with few attempts to address transcriptomic changes across life-cycle stages [1, 25–34]. This means that little is known about the role of genes during its development, reproduction, and across sexes, which may compromise ecological and ecotoxicological interpretations.

*D. magna* has a well-known development and life-cycle stages, which combine parthenogenetic and sexual reproduction [35]. Parthenogenetic reproductive females allocate freshly formed eggs into the brood pouch every 3-4 days at 20 °C, under optimal food conditions [10]. Freshly released eggs develop independently of the mother across 12 visually recognizable embryonic stages into free swimming juveniles [36] that are released by the mother just before it moults and allocates a new clutch of eggs into its brood pouch [10]. Juveniles, which are morphologically similar to adults, moult four or more times, depending on food conditions, before closing the circle by allocating the first clutch of eggs into their brood pouch [10]. In the ovary of a *D. magna* reproductive female co-exist clusters of pre and vitellogenic oocytes [35], the latter being provisioned with yolk during the intermoult interval to form the new clutch of eggs at the end of it, whereas the former will mature and became vitellogenic in the next reproductive cycle. Upon adverse conditions, *D. magna* females produce males whose haploid sperm can fecundate haploid oocytes to form an ephippia containing two sexually produced eggs, a process that can be triggered independently from environmental conditions by exposure to the crustacean juvenile hormone methyl farnesoate [35, 37]. Ephippia eggs can survive for decades in water bodies’ sediments and develop into free-swimming female juveniles when conditions are favourable. Thus *D. magna* is also an excellent model to study large-scale gene transcription across development, reproduction and sexes to provide global relationships between transcription and life-cycle stages.

**Fig. 1:**
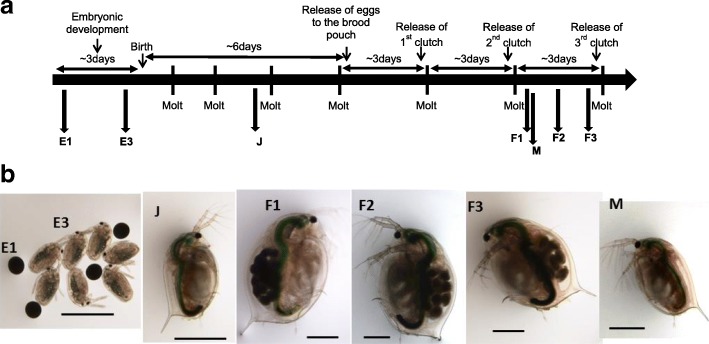
Diagram representing the experimental design. **a** Seven life- stages were selected: embryos < 12 h after oviposition (Eggs 1, E1; corresponding to eggs of stage 1 following [1]), embryos within the last 12 h to be release as free swimming first instar juveniles (Eggs 3, E3; corresponding to embryos of stage 12 according to [1]); juvenile stage (J, animals of 4 days old), females F1, F2, F3 representing third brood females at the beginning, middle and end of their intermoult cycle, respectively. The final stage includes a reproductively active male (M) with 14 days. **b** images of the studied developmental stages, bars in each image are 1 mm. Further details about the description of life-stages are in methods section

Novel developments of bioinformatic tools [38] and of DNA/RNA-seq technologies have allowed the publication of the *Daphnia pulex* full genome [20] and more recently of the full *D. magna* transcriptome [1]. Information reported by Orsini et al. [1] of approximately 42,000 gene validated models, including splicing forms, was used in this present study to design a new microarray, representing the full gene set of the *D. magna* transcriptome coupled with most up-to-date functional information. The new microarray platform was then used to study gene transcription patterns for the full transcriptome of *D. magna* across early and late embryonic stages, juveniles, reproductive females and males (Fig. 1).

## Results

### Microarray performance

The probe design was checked for specificity performing a complete probe match analysis against the *D. magna* original gene models. Using exact match criteria 3616 probes match to two or more transcripts. If allowing for 1-3 mismatches (5% divergence) 5371 of the probes matched to two or more transcripts.

The microarray ability of representing real changes in transcription was checked performing qPCR on four relevant genes from three different pathways (Krebs cycle, tryptophan metabolism and ecdysteroid pathways). The results between the two technological platforms were highly correlated (Pearson correlation > 0.73, *P* < 0.01, *N* = 20, Additional file 1: Figure S1).

### Differentially transcribed genes

**Fig. 2:**
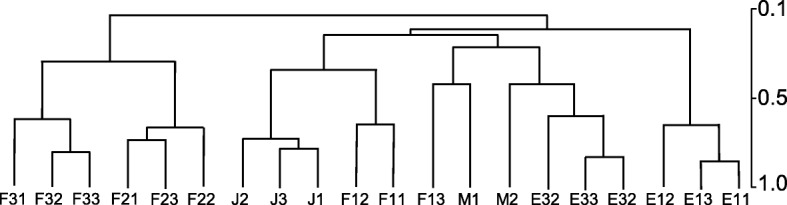
Pearson hierarchical clustering of the studied samples. E1, E3, J, F1, F2, F3 and M refer, respectively, to eggs 1,3, juveniles, females 1,2,3 and males. Second numeral (i.e. E11, E12, E13) refer to replicate 1,2 and 3, respectively

About 109,640 probes showed fluorescence values above the background. These probes belonged to 38,449 unique genes out of the 41,317 *D. magna* genes present in the microarray design (see Additional file 2 for further information regarding the non-expressed genes). Hierarchical clustering of fluorescence values for the 109,640 probes in all samples showed a good grouping of biological replicates (Fig. 2), a sign for good quality of the design. The analysis defined three main clusters: Cluster 1 composed by F2, F3 samples; Cluster 2 grouping E3, M, F 1 and J stages; and Cluster 3 including only E 1 samples.

**Fig. 3:**
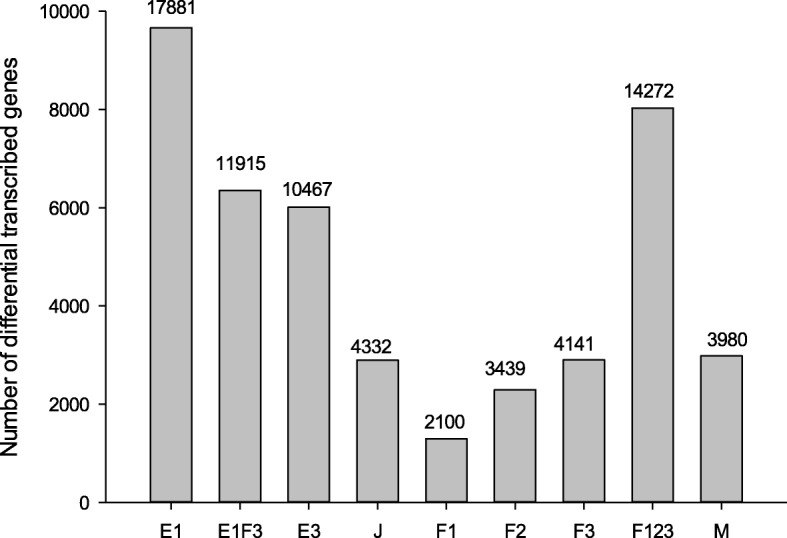
Differentially transcribed genes across the studied *D. magna* life- stages. Numbers above each bar indicates number of probes. E1, E1F3, E3, F1, F2, F3 and F123 means respectively, eggs 1, eggs 1 & females 3, eggs 3, females 1,2 and 3, the combined three female stages (F123). M stands for males

The total number of differentially transcribed genes (DEG) can also be an important measure of how active the cells of an organism are during a particular life stage. The number of probes (DEP), and the unique genes these represent, differentially transcribed across life-stages was quite variable (Fig. 3 and Additional file 1: Tables S1 and S2). The highest number of DEP corresponded to the E1 stage (17881 DEP, representing 9663 unique genes), steadily decreasing to 10467 DEP (6013 Genes) in E3 and 4332 DEP (2893 Gene) in juvenile stages (Fig. 3). F1 females had the lowest number of DE compared to the all other life stages with 2100 DEP (1294 Genes), being that number increased to 3439 DEP (2292 genes) and 4141 (2900 genes) in F2 and F3, respectively. To allow a better comparison of transcriptomic changes across sexes all female stage x replicates were considered together, which totalled 14272 DEP (8026 genes). Furthermore, up to 6352 genes (11915 DEP) were differentially upregulated in both E1 and in F3 (E1F3, Fig. 3). Finally, males differentially expressed a total of 3980 DEP (2983 genes). In most life stages there were more up-regulated DEG than down regulated ones except for F1. Details of the expression levels of differentially transcribed probes and genes at the various studied life-stages are provided in Additional file 3.

**Fig. 4:**
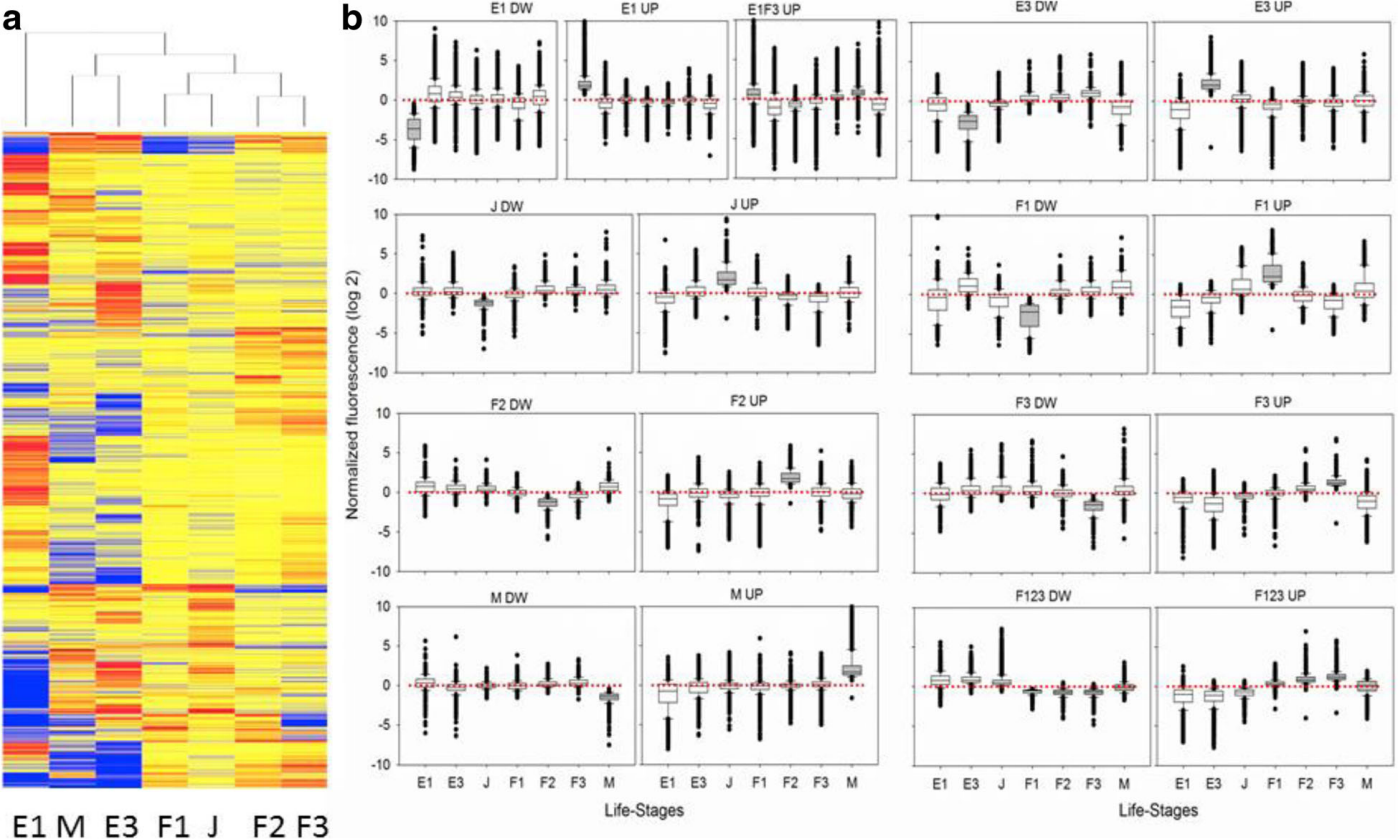
Heat map of differentially transcribed genes clustered across the studied *D. magna* life- stages (**a**) and box-plot of transcription pattern of up and down regulated genes of each life-stage against the rest (**b**). In graph A red, blue and yellow colours indicated up, down and unchanged transcription levels. Grey boxes indicated the reference life-stage. For clarity analyses were conducted on averaged values of experimental replicates

The heat map of DEG included three clusters (Fig. 4a): the first one constituted by E1 samples, a second other including E3 and M samples, and a third cluster grouping the three female stages (J, F1, F2 and F3). Transcription patterns of up and down regulated DEGs in E1, E3, J, F1, F2, F3, F123 and M were stage-specific, whereas that of E1F3UP were up regulated in E1 and F3 (E1F3) and to a lesser extent in F2 and F1.

### Functional analysis

A total of 25,019 (60%) genes present in the microarray were functionally annotated to some level by using the three major databases (Drosophila, REFSEQ and Swissprot KB), being 16,081 genes mapped to at least one GO Term and 7995 annotated to a KEGG-curated gene. Using a high stringency probability (*p* < 0.001), a total of 2905 distinct GO terms were enriched across the different life-cycle stages (Additional file 4), 364 of them (about 12%) being common in at least two life-stages (Table 1). Selected GO terms and their coverage in each stage are shown in Table 2.

**Table 1:** Number of common enriched Gene Ontology terms (GOs) across the studied life stages and in the cluster of genes upregulated in E1 and F3. Further information is in Additional file 4

Life-stages	E1F3	E3	J	F1	F2	F3	F123	M
E1	407	133	0	0	8	15	305	2
E1F3		121	2	4	5	33	75	1
E3			25	18	16	24	190	2
J				8	8	10	6	0
F1					45	7	3	10
F2						10	30	1
F3							27	0
F123								0

**Table 2:** Total and selected significant (*P* < 0.001) enriched Gene Ontology terms (GOs) of the differentially transcribed genes across the different studied life-cycle stages including genes of E1 from maternal origin (E1F3). N, %Cov are number of probes and coverage. Further details are in Additional file 4. Stage abbreviations are explained in text

Acession	GO Term	E1		E1F3		E3		J		F1		F2		F3		F123		M	
Total number		1380		829		1323		97		145		146		91		1004		27	
		N	% Cov	N	%Cov	N	% Cov	N	% Cov	N	% Cov	N	% Cov	N	% Cov	N	% Cov	N	% Cov
GO:0002176	male germ cell proliferation																	9	0.5
GO:1901476	carbohydrate transporter activity													24	1.5			29	1.6
GO:0042302	structural constituent of cuticle					111	2.1			135	13.1	57	3.6	37	2.3			48	2.7
GO:0009838	abscission													10	0.6				
GO:0097493	struct molec act conferring elasticity					83	1.6			97	9.4	51	3.3	30	1.9				
GO:0032993	protein-DNA complex	218	2	93	1.6									39	2.5	107	1.7		
GO:0042445	hormone metabolic process					223	4.2							81	5.1				
GO:0048856	anatomical structure development	7415	68.7			3697	69.8							1128	71.5	4449	70.8		
GO:0044851	hair cycle phase											14	0.9						
GO:0048180	activin complex									5	0.5								
GO:0022611	dormancy process									14	1.4								
GO:0031395	bursicon neuropeptide hormone complex							11	0.6										
GO:0072562	blood microparticle					55	1												
GO:0042562	hormone binding					88	1.7												
GO:0007626	locomotory behavior					337	6.4												
GO:0004872	receptor activity					418	7.9												
GO:0097458	neuron part					1135	21.4									1186	18.9		
GO:0071944	cell periphery					2158	40.8									2529	40.3		
GO:0042221	response to chemical					2366	44.7												
GO:0009653	anatomical structure morphogenesis	4718	43.7	261.5	44.7	2370	44.8									2959	47.1		
GO:0044767	single-organism developmental process	7414	68.7			3683	69.6									4523	72.1		
GO:0002200	somatic diversification of immune receptors	70	0.6																
GO:0002262	myeloid cell homeostasis	172	1.6																
GO:0030496	midbody	217	2	154	2.6														
GO:0002253	activation of immune response	370	3.4																
GO:0032259	methylation	663	6.1	394	6.7														
GO:0000989	transc fact act, transcription factor binding	704	6.5																
GO:0000785	chromatin	783	7.3																
GO:0002520	immune system development	914	8.5																
GO:0008283	cell proliferation	1924	17.8													1174	18.7		
GO:0016265	death	2139	19.8																
GO:0003006	developm proc. involved in reproduction	2267	21																
GO:0048646	anatomical struct form morphogenesis	2681	24.9													1689	26.9		
GO:0044085	cellular component biogenesis	4056	37.6																
GO:0006950	response to stress	4684	43.4													2793	44.5		
GO:0006807	nitrogen compound metabolic process	5825	54	3322	56.8														
GO:0044422	organelle part	6354	58.9																
GO:0016043	cellular component organization	6437	59.7																

**Fig. 5:**
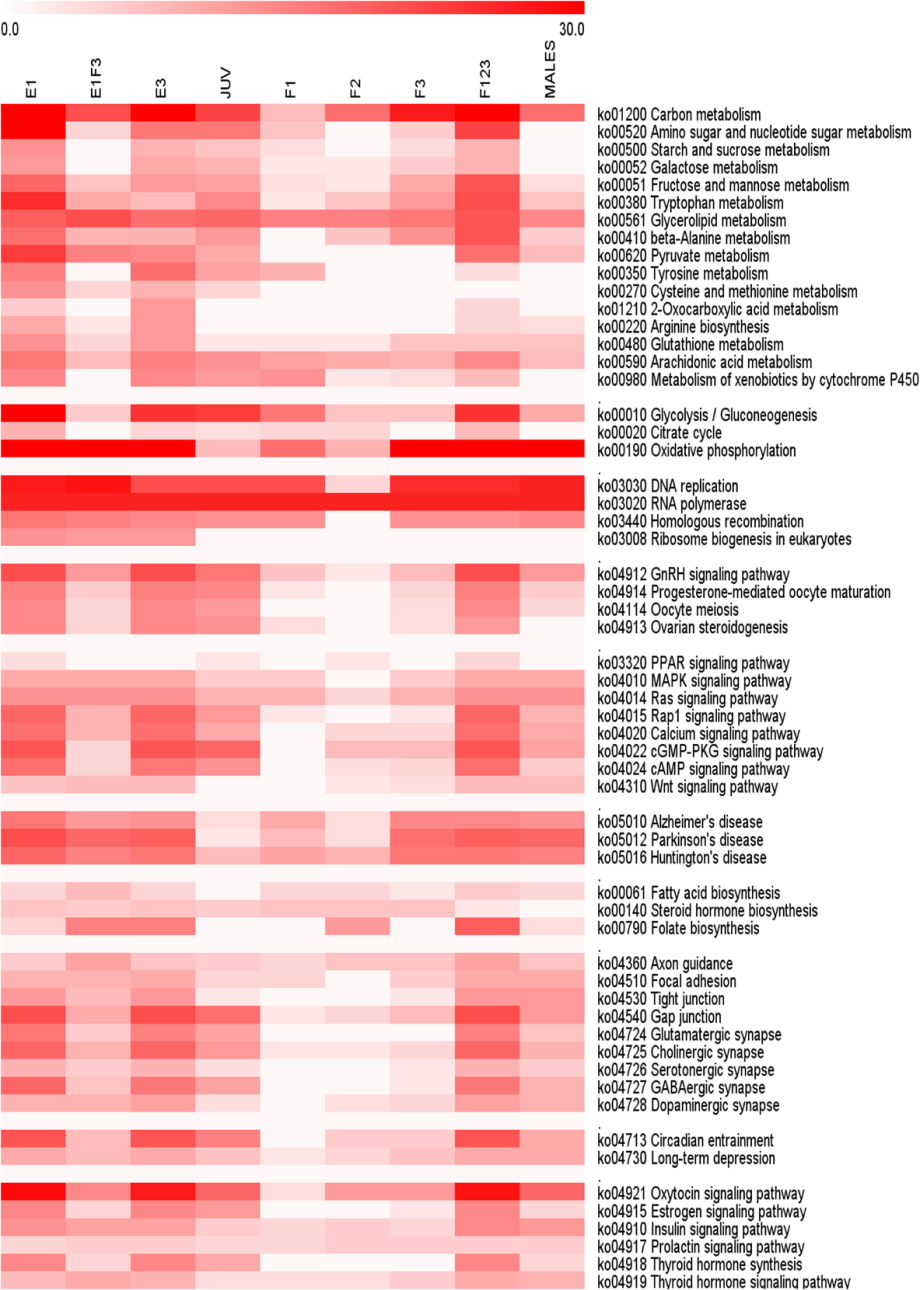
Heat map of the enriched KEGG pathways among the differential transcribed genes in each life-stage and clusters. Scale refers to number of genes in each pathway. Further details can be found in Additional file 5

KEGG mapping of specific DEGs generally agreed with the identified GO terms, but they present a better overview on how the number of differentially expressed genes mapped to specific pathways along the development of the organism (Fig. 5 and Additional file 5).

## Discussion

Most of the probes included in the microarray match a unique gene transcript with only about 3.2% of probes matching two or more gene transcripts. Furthermore correlation values of selected gene transcript levels between qPCR and microarray probes were high, even for low differentially transcribed genes, thus indicating a good quality of the probes, capable of reflecting even small changes in mRNA abundance.

More than 93% of the genes that are reported to be transcribed in *D. magna* genome [1] showed fluorescence values above background levels in at least one out of the seven studied life-cycle stages. Hierarchical clustering of samples and the number of differentially transcribed genes (Figs. 2 and 3) separated eggs from stage E1 that showed the greatest levels of differential transcribed genes (34.5% of the 38,449 genes), reproductive females of stage F2 and F3 with up to 18.5% of the transcribed genes and the rest of life stages (E3, J, F1 and M) with the remaining 47% of the gene transcripts. These three clusters had distinctive developmental/physiological characteristics. *D. magna* females transfer large amounts of resources including mRNA to their eggs to ensure their development and survival upon hatching [28, 39–42]. Therefore, E1 is likely to be enriched with genes associated to embryonic development and maternal transfer. Indeed up to 6352 genes were up-regulated in E1 and in females F3 (E1F3 group in Fig. 3). This is in line with reported transcriptomic results in *D. melanogaster*, *Anopheles* and *C. elegans*, which found that the majority of genes were transcribed during early embryogenesis stages due to the contribution of both maternal and zygote ones [2–5]. The second cluster, grouping F2 and F3 females, can be considered representative of the most reproductive active stages, whereas life-stages included the third cluster (E3, J, F1 and M) are associated to high moulting activity and low-reproduction related processes [43, 44]. The relative low levels of differentially transcribed genes in F1 females (4.6% relative to the total), combined with an overrepresentation of down-regulated genes (see Additional file 1: Table S2) can be related to the fact that F1 organisms perform a reset of their metabolic and physiological processes at the beginning of the intermoult cycle [43, 44]. Therefore, most of the specific differences showed in this stage could be related to de-activation of gene transcription.

Gene transcription across the studied life-stages (Fig. 4) also evidenced clear distinctive patterns across egg, juvenile and adult stages. Up regulated DEGs in reproductive females and males showed low transcription levels in the rest of life-stages whereas low transcribed ones were highly transcribed in the remaining stages. In the embryonic stage E3 and juveniles, genes having high or low levels of transcription changed little in the remaining stages. The early embryonic stage E1 showed two distinctive transcription patterns: a cluster of 9663 unique genes highly or poorly transcribed in that stage and a cluster of 6352 genes highly transcribed in that stage and in female stages F3 and F2. These results agree with those reported in *Drosophila* and *C. elegans* supporting the argument that many transcripts in E1 have a maternal origin [2, 3]. The unique gene patterns identified in most life-stages and across sexes also support previous studies that associate these stages with specific subsets of genes being expressed in specific tissues and biological processes [2, 3].

### Gene function analysis

E1 transcriptome included the highest number of significant GO terms (1380), mainly in categories related to division and proliferation, morphogenesis, homeostasis, and immune response (Table 2). Homeostasis is considered important to cells on high metabolic rates in which accumulation of some metabolites can become toxic. The enrichment in methylation-related transcripts may be linked to epigenetic processes, which are common during embryo development in Daphnia and other arthropods [2, 28, 45]. Interestingly the cluster of genes from E1 coming from a maternal origin (E1F3) were enriched with 829 GO terms, 50% of them also present in E1 (Tables 1 and 2). Transcripts enriched in E3, with up to 1323 significant GO terms, also had important representation of GOs related with morphogenesis/organogenesis. They also include several GOs related to response to chemicals (Table 2), perhaps preparing the organism to survive in the outside environment [46]. This stage also show an enrichment of GO terms related with sensorial and locomotion processes (neuronal activation and differentiation, hormonal metabolic processes and binding and locomotor behaviour), which would prepare the embryo for the free-swimming stage. The E3 transcriptome was also enriched in GOs related to cuticle constituents and elasticity, a functional category common to all the adult female stages. Transcriptome from the juvenile stage was annotated to 97 significantly enriched GO terms (Additional file 4), including the distinctive GO term “Bursicon neuropeptide hormone complex” (Table 2), which is related to the hardening of carapace [47]. This is in line with the fact that moulting and growth processes dominate in this stage over other biological functions such as reproduction [48]. Enriched GO terms from F1 transcriptome (145 GO terms in total) included two distinctive GO’s: dormancy process and activin complex. The first GO term is possibly related to the re-setting of moulting and reproductive processes in which most bodily functions are “on hold” [49]. The activin complex GO term is directly related with the Follicle Stimulating Hormone (FSH) a key hormone to trigger the development of ovules [50]. This is a key process of extreme importance to the reproduction of these organisms showing that any impairment of these genes during this life stage period can be critical for the reproductive output. This is consistent with the fact that ovulation occurs precisely at the F1 stage [35]. In F2, specific transcripts were annotated to 146 significant GO terms, with one distinctive term, hair cycle phase, which can be linked in these organism to the formation of carapace and, possibly, also to hardening processes. Analysis of the F3 transcriptome identified 91 enriched GO terms, two of them with a high coverage (47%, 71% coverage of the annotated probes DEG in this stage) and belonging to “Anatomical Structure Development or morphogenesis”. These terms may be related to two processes: (1) the development of the ovary and formation and provisioning of eggs and (2) the maternal transfer of RNAs from the mother to the newly formed eggs to start development. Interestingly, a large fraction of probes annotated to Egg stages and of maternal origin (E1F3) also belong to this GO term. Another key term uniquely related to F3 was “Abcission”, which in arthropods is involved in carapace shedding [51], a key event in the development of crustaceans that are only able to grow by periodically releasing the old carapace and the subsequent formation of a new one. This last GO term is likely related to the fact that females F3 are close to a moult event. Differentially transcribed genes of reproductive females (F123) belonged to 1004 GOs. Almost half (444) of those GO also existed in E1 and E3. Male-specific transcripts were annotated to 97 GO terms, including the unique category of “Male Germ Cell proliferation”, which in turn includes the gene *doublesex*, linked in *Daphnia* to male sexual differentiation [52]. Males also present a high number of probes associated with carbohydrate transport activity, like F3, which can related to a high demand of energy for swimming in males [53] and to reproductive activities for females [48].

The metabolism-oriented KEGG pathway mapping (Fig. 5 and Additional file 5) shows a high number of DEG related to carbon, starch and sucrose, tryptophan and fatty acid metabolism in embryo (E1, E3 and juvenile (J) stages, while these numbers were reduced in adult males (M) and females (F1, F2, F3). Interestingly genes of E1 coming from a maternal origin were less enriched in the above mentioned metabolic pathway. Within the adult stages, F3 had the greatest number of DEGs belonging to KEGG metabolic related pathways, likely related to the animal needs to synthesize a new carapace and to finalize egg provisioning, both processes having a great demand of energy.

DEGs related to DNA replication were abundant in all life stages and in the cluster of E1 from maternal origin (E1F3) with except for F2. In the parthenogenically-reproducing sister species *Daphnia pulex*, RNA and DNA concentrations were related to changes in metabolic activity associated with moulting cycle and ontogenetic development [54]. Ontogenic developmental processes are likely to dominate in embryo stages (E1, E3). Females F2 are just in the middle of the intermoult cycle, whereas the rest of free living stages (J, F1, F3) are likely to be just at the beginning or end of the moulting cycle.

An important point to consider when evaluating the quality of the data we are reporting of its functional analysis is the fact that males have no DEG mapped on females processes and hormones (Gonadotropin, progesterone, oocyte and ovarian steroidogenesis). All these processes were highly enriched in eggs, juveniles and F1,2,3.

Embryonic stages showed a high expression of genes annotated to many signalling pathways, like MAPK (response to stress), RAS (cellular signal transduction), RAP (vital for effective signal transduction), cAMP (second messenger important in several biological processes), and Wnt (signal transduction pathways made of proteins, extremely important for embryonic development). Genetic and molecular studies in *Drosophila melanogaster* over the last several years show that these signalling pathways are functionally conserved and that they participate in numerous processes during normal development [55–59].

Similarly, genes implicated in neuronal processes were highly expressed in egg and female stages. This is in line with the development of the neuronal system in metazoans along the embryonic stages [60]. Finally, hormonal processes related to Prolactin and insulin were evenly differentiated across life cycle stages, whereas genes belonging to thyroid hormone synthesis and oxytocin KEGG pathway, were differentially regulated in egg and female stages. Despite that genes and peptides of the above signalling pathways have been found in *D. pulex* and *D. magna* genome, little is known about their expression and function [61–63].

## Conclusions

In this work we present a new microarray platform designed using the full set of gene models representing the complete genome of *D. magna* [1]. Up to 93% of the existing 41,317 *D. magna* gene models showed differential transcription patterns across the developmental stages of *D. magna*, from which more than 59% were functionally annotated. The transcriptome analysis show a large number of embryo-specific transcripts, likely related to the high metabolic rates needed for cellular multiplication and morphogenesis of several body organs, as inferred by the high number of DEG related to DNA, RNA and ribosome biogenesis. In adult females, most DEG were involved in reproductive processes and carapace shedding. Differentially transcribed genes in males were enriched in specific genes of male sexual differentiation genes, like *doublesex* [52]; consistently, they did not include hormonal female-related functional categories. These results are in line with those reported in *Drosophila,* showing that about 90% of transcription of embryo-specific transcripts occurs during the first hours of embryo development [2].

## Methods

### Experimental animals and culture conditions

All experiments were performed using a well-characterized single clone of *D. magna* (Clone F), maintained indefinitely as pure parthenogenetic cultures. Individual or bulk cultures of 10 animals/L were maintained in ASTM hard synthetic water [64] as described in [44]. Individual or bulk cultures were fed daily with *Chorella vulgaris* Beijerinck (5 × 10^5^ cells/mL, respectively, corresponding to 1.8 μg C/mL; [10]). *C. vulgaris* was grown axenically in Jaworski/*Euglena gracilis* 1: 1 medium (CCAP, 1989). Algae were harvested in the exponential phase of growth, centrifuged and then re-suspended in ASTM hard water. The number of algal cells in freshly prepared medium was checked daily from absorbance measurements at λ = 650 nm in a dual-beam spectrophotometer (Uvikon 941) using standard calibration curves based on at least 20 data points, with an r^2^ > 0.98. The culture medium was changed every day, and neonates were removed within 24 h. Photoperiod was set to 14 h light: 10 h dark cycle and temperature at 20 ± 1 °C.

For this study, females across six different developmental stages and one adult male stage were collected in triplicate. Embryonic samples were taken according to the embryonic development staging works by Mittmann et al. [36], while the free swimming stages were sampled following major physiological events. A graphic representation of these stages is depicted in Fig. 1. The first egg stage (E1) includes embryos < 6-8 h after ovideposition in stage 1 [36]. At Stage 1 the nucleus lies within non-transparent globular pale to greenish yolk granules and several larger oil drops. The egg is covered tightly by the chorion. The second embryonic stage selected (E3) corresponds roughly to stage 12 [36] and includes embryos within their last 12 h before the release from the mothers brood pouch. At this stage the embryonic development is mostly completed and is in the path of transformation to the first instar juvenile [36], being already able to swim. The choice of this stage was made to represent a neonate without the interference of feeding behaviour and or digestion processes. The third selected stage (J) is a fourth instar juvenile or pre-adolescent stage. Animals of about 4 days old (with no ovary visible) [65], targeted to represent a full working organism but without the interference of reproductive processes.

Adult third clutch female stages (F1), (F2) and (F3) represent, respectively, reproductive females at the beginning < 12 h), middle (30 h < X < 40 h) and end (> 66 h) of their reproductive/intermoult cycle, which usually lasts 3 days at 20 °C with non-limiting food conditions as in the present study [44]. Females F1 were collected just after moult and release of the new clutch of newly formed eggs of stage 1 (i.e. E1 embryos) into their brood pouch (< 8 h). Females F2 were sampled after approximately 36 h and F3 were collected within the last 24 h before moulting and releasing fully developed embryos. Females F3 thus hold embryos of stage 12 (our E3 embryos). Ovulation and egg provisioning occurs simultaneously during a single reproductive/intermoult cycle [35]. In females F1 pre-vitellogenic eggs pass to vitellogenic ones in the ovary and starts the egg provisioning, which is fully accomplished in F3. The development of embryos in the brood pouch also occurs simultaneously as ovulation and egg provisioning [35]. Females were de-brooded before being collected, by gently flushing water into the brood pouch. Removing the developing embryos from the brood pouch also removes their RNA, allowing for a much more robust profiling of the female adult stages [10]. The final samples include reproductively active males. These males were obtained exposing parthenogenic females to 0.5 μM of methyl farnesoate [37]. After birth, the males were selected observing the size of the first antenna and let to grow until 14 days, representing a physiological status roughly coinciding with female stages.

### RNA extraction

Total RNA was isolated from the samples using Trizol reagent (Invitrogen, USA) and following manufacturer protocols with slight modifications. After RNA isolation, DNAse treatment was performed according to manufacturer protocols, followed by a double phenol-chloroform and another chloroform extraction for further purification. RNA was precipitated using Sodium acetate and 100% ethanol, re-suspended in RNAse free water being quantity and quality checked using a NanoDrop D-1000 Spectrophotometer (NanoDrop Technologies, USA). Samples presenting a ratio 230/260-260/280 between 1.9-2.1 were selected. RNA integrity was checked using an Agilent 2100 Bioanalyzer (Agilent Technologies, USA). Only the samples showing invertebrate-adjusted RIN values above 9 were used for microarray analysis.

### Microarrays

This new design was made using the gene models developed by Orsini et al. [1]. This gene set includes 41,317 gene models representing the full transcriptome of *Daphnia magna*. Four probes were designed for every gene, using the algorithms available in the e-array tool from Agilent (Palo Alto, USA). One of the probes was the best possible probe, while the other three were designed to represent a best geographical representation of the gene sequence, i. e., one probe representing the beginning of the sequence, another one representing the central part and the last the end part of the sequence. After probe design, redundancy was checked and in case of existence, redundant ones were removed. A set of 3500 random computer-generated probes were also added as further negative controls. Further e-array based quality controls were added, resulting in a microarray with 165,000 probes. This was then printed on a 4 × 180,000 format (Agilent 66414 design; GPL22721). A total of three replicates per treatment were used. One μg of total RNA was used for all hybridizations. cDNA synthesis, cRNA labelling, amplification, and hybridizations were performed following the manufacturer’s kits and protocols (Quick Amp labelling kit; Agilent, Palo Alto, CA). The Agilent one-color Microarray Based Gene Expression Analysis v6.5 was used for microarray hybridizations according to the manufacturer’s recommendations. Microarray images were generated by an Agilent high-resolution C microarray scanner. Data was resolved from microarray images using Agilent Feature Extraction software v10.7. Raw microarray data from this study have been deposited at the Gene Expression Omnibus Web site (www.ncbi.nlm.nih.gov/geo/) with accession number GSE90810.

### Gene expression analysis

Microarray data were analysed using Gene Spring GX v13.0 software (Agilent, USA). Fluorescence data were normalized using quantile normalization and baseline transformation to the median of all samples.

The quantile 95 of the 3500 negative probes was calculated and this value was assumed as being the fluorescence background noise value of each sample. Only the probes having fluorescence values above background noise values (109,640 probes in total) were considered for further analyses. Sample Clustering of the total expressed genes across life-stages was analysed using the Multi-Experiment viewer MeV4 software [66] by hierarchical clustering using Pearson correlation algorithm.

Differentially transcribed probes (DEP) of *D. magna* individuals in a given life-stage were identified by pairwise comparison of the normalized replicated fluorescent levels of that stage against the other stages using Student t test *p* < 0.001) and a fluorescence change cut off of 1.5-fold. The later term was calculated as the quotient between the power of two of the mean normalized fluorescence of both stages involved in the comparison. Only those probes whose normalized fluorescence changed significantly ≥1.5 fold in all pairwise “stage” vs “all others” comparisons were considered. For a given stage probes having greater or lower normalized fluorescence values versus the rest of stages were considered up and down regulated. This approach was found to be more conservative than the combination of ANOVA approaches and the Benjamini-Hochberg false discovery rates correction. Finally, differentially transcribed genes (DEG) was defined as a non-redundant representation of the genes uniquely represented by at least one DEP. To determine genes present in E1 from maternal origin, differentially transcribes probes upregulated in both E1 and F3 were determined by comparing the normalized replicated fluorescent levels of both stages against the juvenile stage using Student t test *p* < 0.001. Stages F3 and J were selected since F3 is the female stage where more resources from ovaries are allocated to eggs, whereas J can be considered an immature female stage [10].

### Validation of microarray results by qPCR

Microarray results were validated with real-time quantitative polymerase chain reaction (qPCR). We selected four differentially expressed genes from different pathways/gene families which had already been successfully used in previous studies [46, 67]: Krebs cycle (isocytrate dehydrogenase, idh; ATP citrate lyase, ATPCL), tryptophan metabolism (dopamine decarboxylase, Ddc) and Ecdysteroid pathways (Ecdysone receptor, EcR b). The G3PDH gene (glyceraldehyde 3-phosphate dehydrogenase) was used as internal control (Housekeeping). Primers for each one of these genes were designed with Primer Express® Software v3.0.1(Thermofisher, USA) and are provided elsewhere [46, 67]. qPCR was performed according to manufacturer’s protocols.

### Gene model annotation

For the optimal interpretation of transcriptional changes it is fundamental to have a functional annotation of all genes. To achieve this, all the 41,317 genes were searched for homology and functional annotation, including Gene Ontology (GO) and Kyoto Encyclopaedia of Genes and Genomes (KEGG). This was performed using Blast2Go Suit [26]. To maximize homology findings, this search was performed using BLASTX alignment algorithms (search protein databases against a translated nucleotide query). Search was performed using as target the three main databases of interest to our platform: Drosophila, RefSEQ and SwissprotKB. The three annotation sets were finally merged. The ultimate objective of re-annotation of the gene models was to improve as much as possible the functional annotation of these. This is the reason why we limited our search to the reference databases as these are the ones with most functional mapping. The full details of the annotation are available in GEO together with the microarray design (GPL22721).

## Additional files

Additional file 1: Word file with the qPCR validation of the results in Figure S1 and the total number probes and genes of a given life-stage differential transcribed across the remaining ones and those up and down regulated in Tables S1 and S2 (DOCX 40 kb)

Additional file 2: Excel file with specific genes which were not transcribed (Fluorescence below background) throughout the development stages. (XLSX 89 kb)

Additional file 3: Excel file with specific probes and genes differentially transcribed across life-stages. (XLSX 26161 kb)

Additional file 4: Excel file with GO terms enriched across the different life-cycle stages (XLSX 411 kb)

Additional file 5: Excel file with KEGG terms across the different life-cycle stages (XLSX 5169 kb)

## Abbreviations

DEG: Differentially expressed genes
DEP: Differentially expressed probes
E1: Egg in stage 1
E3: Egg in stage 3
F1, F2, F3: Reproductive females in stages 1, 2 and 3 respectively
F123: Combinations of females 1, 2, 3
