# Supplementary material for: Differential gene transcription across the life cycle in Daphnia magna using a new all genome custom-made microarray
Source: BMC Genomics. 2018 May 18;19:370. doi: 10.1186/s12864-018-4725-7 (PMC5960145; doi:10.1186/s12864-018-4725-7)
Supplement: Supplementary file 1 — Word file with the qPCR validation of the results in Figure S1 and the total number probes and genes of a given life-stage differential transcribed across the remaining ones and those up and down regulated in Tables S1 and S2 (DOCX 40 kb) [file 12864_2018_4725_MOESM1_ESM.docx]

**Figure S1**. Confirmation of the array results with qPCR. Y axis represents the qRT-PCR results in gene copy number per 1000 copies of the house keeping gene G3PDH. X axis represents the log2 fluorescence values after normalization. Errors bars are standard errors of the mean (SE, *N* = 3).

Table S1. Number of probes (DEP) of a life-stage differentially transcribed across the rest of life-stages. Within a given life-stage left and right numbers are up and down regulated probes, respectively. Probes are depicted in Additional file 2.

| Total | Life-stage | E1 | | E 3 | | J | | F1 | | F2 | | F3 | | M | |
| --- | --- | --- | --- | --- | --- | --- | --- | --- | --- | --- | --- | --- | --- | --- | --- |
| 17881 | E1 |  |  | 11323 | 6558 | 11201 | 6680 | 11253 | 6628 | 11196 | 6685 | 11239 | 6642 | 11188 | 6693 |
| 10467 | E3 | 6221 | 4246 |  |  | 6284 | 4183 | 6315 | 4152 | 6254 | 4213 | 6288 | 4179 | 6438 | 4029 |
| 4332 | J | 3338 | 994 | 2679 | 1653 |  |  | 3343 | 989 | 3328 | 1004 | 3353 | 979 | 3235 | 1097 |
| 2100 | F1 | 1374 | 726 | 961 | 1139 | 916 | 1184 |  |  | 826 | 1274 | 946 | 1154 | 958 | 1142 |
| 3439 | F2 | 2555 | 884 | 2221 | 1219 | 2384 | 1056 | 2340 | 1100 |  |  | 2065 | 1375 | 2335 | 1105 |
| 4141 | F 3 | 3154 | 987 | 2770 | 1371 | 2893 | 1248 | 2901 | 1240 | 2852 | 1289 |  |  | 2750 | 1391 |
| 3980 | M | 2410 | 1570 | 2687 | 1293 | 2428 | 1552 | 2415 | 1565 | 2449 | 1531 | 2448 | 1532 |  |  |

Table S2. Number of genes (DEG) of a life-stage differentially transcribed across the rest of life-stages. Within a given life-stage left and right numbers are up and down regulated genes, respectively. Genes are depicted in Additional file 2.

| Total |  | E1 | | E3 | | J | | F1 | | F 2 | | F 3 | | M | |
| --- | --- | --- | --- | --- | --- | --- | --- | --- | --- | --- | --- | --- | --- | --- | --- |
| 9663 | E1 |  |  | 5740 | 3923 | 5651 | 4012 | 5699 | 3964 | 5676 | 3987 | 5679 | 3984 | 5650 | 4013 |
| 6013 | E3 | 3258 | 2755 |  |  | 3279 | 2734 | 3294 | 2719 | 3261 | 2752 | 3277 | 2736 | 3413 | 2600 |
| 2893 | J | 2051 | 842 | 1698 | 1195 |  |  | 2054 | 839 | 2016 | 877 | 2061 | 832 | 2008 | 885 |
| 1294 | F1 | 811 | 483 | 533 | 761 | 546 | 748 |  |  | 457 | 837 | 542 | 752 | 548 | 746 |
| 2292 | F2 | 1628 | 664 | 1419 | 873 | 1514 | 778 | 1490 | 802 |  |  | 1264 | 1028 | 1494 | 798 |
| 2900 | F 3 | 2182 | 718 | 1906 | 994 | 2001 | 899 | 2006 | 894 | 1965 | 935 |  |  | 1910 | 990 |
| 2982 | M | 1598 | 1384 | 1845 | 1137 | 1635 | 1347 | 1630 | 1352 | 1640 | 1342 | 1672 | 1310 |  |  |
